# Improving Facility Performance in Infectious Disease Care in Uganda: A Mixed Design Study with Pre/Post and Cluster Randomized Trial Components

**DOI:** 10.1371/journal.pone.0103017

**Published:** 2014-08-18

**Authors:** Marcia R. Weaver, Sarah M. Burnett, Ian Crozier, Stephen N. Kinoti, Ibrahim Kirunda, Martin K. Mbonye, Sarah Naikoba, Allan Ronald, Timothy Rubashembusya, Stella Zawedde, Kelly S. Willis

**Affiliations:** 1 Departments of Global Health and Health Services, University of Washington, Seattle Washington, United States of America; 2 Accordia Global Health Foundation, Washington, District of Columbia, United States of America, and Department of Epidemiology and Social Medicine, University of Antwerp, Antwerp, Belgium; 3 Accordia Global Health Foundation, Washington, District of Columbia, United States of America; 4 Center for Human Services, University Research Co. LLC, Bethesda, Maryland, United States of America, and Fio Corporation, Toronto, Ontario, Canada; 5 Elizabeth Glaser Pediatric AIDS Foundation, Mbarara, Uganda; 6 Infectious Diseases Institute, Makerere University, Kampala, Uganda and Department of Epidemiology and Social Medicine, University of Antwerp, Antwerp, Belgium; 7 Department of Medicine, University of Manitoba, Winnipeg, Manitoba, Canada; 8 Infectious Diseases Institute, Makerere University, Kampala, Uganda and Institute of Development Policy and Management, University of Manchester, Manchester, England; 9 Infectious Diseases Institute, Makerere University, Kampala, Uganda; University of Ottawa, Canada

## Abstract

**Background:**

The effects of two interventions, Integrated Management of Infectious Disease (IMID) training program and On-Site Support (OSS), were tested on 23 facility performance indicators for emergency triage assessment and treatment (ETAT), malaria, pneumonia, tuberculosis, and HIV.

**Methods:**

The trial was implemented in 36 primary care facilities in Uganda. From April 2010, two mid-level practitioners per facility participated in IMID training. Eighteen of 36 facilities were randomly assigned to Arm A, and received OSS in 2010 (nine monthly two-day sessions); 18 facilities assigned to Arm B did not receive OSS in 2010. Data were collected from Nov 2009 to Dec 2010 using a revised Ministry of Health outpatient medical form and nine registers. We analyzed the effect of IMID training alone by measuring changes before and during IMID training in Arm B, the combined effect of IMID training and OSS by measuring changes in Arm A, and the incremental effect of OSS by comparing changes across Arms A and B.

**Results:**

IMID training was associated with statistically significant improvement in three indicators: outpatients triaged (adjusted relative risks (aRR) = 1.29, 99%CI = 1.01,1.64), emergency and priority patients admitted, detained, or referred (aRR = 1.59, 99%CI = 1.04,2.44), and pneumonia suspects assessed (aRR = 2.31, 99%CI = 1.50,3.55). IMID training and OSS combined was associated with improvements in six indicators: three ETAT indicators (outpatients triaged (aRR = 2.03, 99%CI = 1.13,3.64), emergency and priority patients admitted, detained or referred (aRR = 3.03, 99%CI = 1.40,6.56), and emergency patients receiving at least one appropriate treatment (aRR = 1.77, 99%CI = 1.10,2.84)); two malaria indicators (malaria cases receiving appropriate antimalarial (aRR = 1.50, 99%CI = 1.04,2.17), and patients with negative malaria test results prescribed antimalarial (aRR = 0.67, 99%CI = 0.46,0.97)); and enrollment in HIV care (aRR = 1.58, 99%CI = 1.32,1.89). OSS was associated with incremental improvement in emergency patients receiving at least one appropriate treatment (adjusted ratio of RR = 1.84,99%CI = 1.09,3.12).

**Conclusion:**

The trial showed that the OSS intervention significantly improved performance in one of 23 facility indicators.

## Background

After several years of the successful expansion of vertical programs for human immunodeficiency virus (HIV), tuberculosis (TB), and malaria, there is growing interest in integration of programs [1]. One of seven principles of the United States' Global Health Initiative is to “increase impact through strategic coordination and integration.” [2] One of four principles of the United Nations Program on HIV/AIDS' Countdown to Zero initiative is “leveraging synergies, linkages and integration for improved sustainability.” [3] One of nine principles of the joint World Health Organization/United Nations Program on AIDS' Treatment 2.0 framework is “decentralization and integration.” [4] A recent Cochrane review however, did not identify any randomized controlled trials of interventions to integrate HIV services with other services for women and children [5].

The World Health Organization has led in creating integrated training programs, including the Integrated Management of Childhood Illness [6]–[10] and the Integrated Management of Adult Illness [11]. The evaluation of the Integrated Management of Childhood Illness training program in Uganda emphasized the effects of supervision visits on quality of care [10] and contributed to increasing interest in educational outreach interventions. A recent Cochrane review did not identify any randomized controlled trials of educational outreach in Africa [12], but two recent trials of educational outreach in South Africa showed encouraging results for TB case detection [13] and antiretroviral treatment [14]. Educational outreach trips to facilities however, can be expensive and potentially duplicative if each vertical program conducts them independently.

For the Integrated Infectious Disease Capacity-Building Evaluation (IDCAP), the IDCAP curriculum development team designed two interventions to improve infectious disease care at primary care facilities: the Integrated Management of Infectious Disease (IMID) training program and On-Site Support (OSS). OSS was a series of nine monthly visits to health facilities with both educational outreach and continuous quality improvement (CQI) activities. The effects of the interventions were measured on the clinical competence [15] and practice of individual clinicians, on 23 indicators of facility performance, and on the population-based mortality of children aged under five years. The acronyms used in this article are presented in Table 1.

**Table 1:** Acronyms.

Acronym	Full name
AFB	Acid-fast bacilli
ART	Antiretroviral therapy
CI	Confidence interval
CQI	Continuous Quality Improvement
ETAT	Emergency Triage, Assessment and Treatment
FLEI	Facility-Level Evaluation Indicator
HIV	Human Immunodeficiency Syndrome
IDCAP	Integrated Infectious Disease Capacity Building Evaluation
IMID	Integrated Management of Infectious Disease
MF5	Medical Form 5
MOH	Ministry of Health
NTLP	National Tuberculosis and Leprosy Program
OSS	On-site support
RR, aRR	Relative risk, adjusted relative risk
RRR, aRRR	Ratio of relative risks, adjusted ratio of relative risks
TB	Tuberculosis

This article contributes an overview of results on all performance indicators for which valid trial data were available. In addition, it reports ancillary analyses of the effects on Arm A sites that selected particular indicators as the focus of their CQI activities compared to Arm A sites that selected other indicators. Expanded analyses of the Emergency Triage, Assessment and Treatment indicators were reported in (Kinoti et al, unpublished manuscript) and the fever and malaria case management indicators were reported in Mbonye et al. [16] Breslow recommended using caution when promoting positive results from studies with multiple outcomes [17], and this overview presents the ETAT and malaria case management results in the context of all available performance indicators.

## Methods

### Ethical Consideration

IDCAP was reviewed and approved by the Uganda National Council on Science and Technology (reference number HS-722) and the School of Medicine Research and Ethics Committee of Makerere University (reference number 2009–175). The data sources were the Uganda Ministry of Health (MOH) forms and registers, so data collection was ongoing at the facilities before the committees' approvals and after IDCAP ended. The IDCAP proposal was for extracting and entering the MOH data, which began after the ethical approvals. Written informed consent was obtained from IMID participants for secondary analysis of the Infectious Diseases Institute training program data [15]. Informed consent of staff at the facilities was not required because the facility performance data were used to evaluate facility rather than individual performance. Informed consent of patients was waived for the indicators reported in this article. The University of Washington Human Subjects Division determined that this study did not meet the regulatory definition of research under 45 CFR 46.102(d).

### Study Design

The objective of the primary analysis of the 23 performance indicators was to test the effects of the IMID training and OSS on ETAT, and care for malaria, pneumonia, TB, and HIV. The evaluation design was mixed with a pre/post component to measure the effect of IMID training alone in Arm B and the combination of IMID training and OSS in Arm A, and a cluster randomized trial component to measure the additional effect of OSS as the difference between the effects in the two arms. Thirty-six facilities were randomized as clusters and the facility performance indicators were analyzed as clusters, because many of the performance indicators depended on a team of clinicians, laboratory professionals and data entry staff rather than individuals. Naikoba et al. [18] summarized the protocol. The protocol for this trial and supporting CONSORT checklist are available as supporting information; see Checklist S1 and Protocol S1. The anonymous data underlying the findings are available on the Global Health Data Exchange website at http://ghdx.healthdata.org/.

### Participants and eligibility

The sites were 36 health center IV or comparable facilities drawn from all regions of Uganda. A health center IV is a referral facility and the highest of four levels of public health facilities, where a health center I is a village health team [19]. Two mid-level practitioners, either clinical officers, registered nurses, or registered midwives, from each of the 36 facilities participated in IMID training. See Miceli et al. [20] and Naikoba et al. [18] for the inclusion criteria for the facilities and clinicians. To isolate the effects of OSS, only facilities that were not actively participating in other quality improvement programs for HIV/AIDS services were included. Among 214 facilities [19], only 38 met this criterion, because two quality improvement programs for HIV/AIDS services were being scaled-up to scores of facilities in Uganda when the sites were selected.

All staff at the facilities were invited to participate in OSS, and patients participated as part of normal clinical activities.

#### Interventions

The IMID training program for mid-level practitioners was taught at the Infectious Diseases Institute in Kampala over the course of five weeks plus distance learning, as described in Miceli et al. [20] The five weeks included a three-week core course and two one-week boost courses 12 and 24 weeks after the core course. The content of many sessions was based on clinical decision guides that were given to all participants as job aids.

OSS was a two-day visit to health facilities once a month for nine months by a mobile team as described by Miceli et al. [20] and Naikoba et al. [18] Briefly, the four mobile team members were a medical officer, a clinical officer, a laboratory technologist, and a registered nurse. Each visit focused on a specific topic and included a follow-up on earlier visits with four types of activities: multidisciplinary team training, one-on-one mentoring, break-out sessions, and CQI activities. To mentor participants, the faculty worked side-by-side with them in the clinic or the laboratory, as relevant, and offered advice or feedback on their practice. Three break-out sessions per topic were organized for three groups of professionals: 1) medical officers, clinical officers, and registered nurses; 2) enrolled nurses, midwives and counselors; and 3) laboratory professionals. Each break-out session was based on a job aid that was given to participants. The break-out training sessions for clinicians were based on the clinical decision guides relevant for each month's topic. For laboratory professionals, they were adapted from the Infectious Diseases Institute's “HIV Laboratory Techniques and Good Laboratory Practices” training to include laboratory techniques for malaria and tuberculosis tests.

The CQI activities were based on the Institute for Health Improvement's collaborative improvement model [21] with adaptations for low and middle income countries. They focused on 13 goals that were associated with a subset of 13 of the 23 facility performance indicators, as reported in Naikoba et al. [18] Each site was asked to select six of the 13 goals and associated indicators, which were called “Facility-Level Evaluation Indicators” (FLEI), because CQI was based on the philosophy that facility teams were more motivated when they selected their goals. The facility teams created or adopted the processes of care to reach the goals they selected. The processes were not at the discretion of the investigators. Facility teams potentially created or adopted more effective processes, because they were most familiar with their work environment. Facility CQI teams attended two Learning Sessions in August and November 2010 to share innovations among Arm A facilities.

### Outcomes

#### Variable definitions and data sources

The 23 facility performance indicators are presented in Table 2. The definitions are in Table S1, which reports the numerator, denominator, data source, and any revisions from the protocol. The indicators were selected according to the following three criteria: 1) the topic was covered in the IMID curriculum, 2) the data were available from MOH forms and registers, and 3) the indicator was linked to outcomes in published cost-effectiveness analyses that would be used in the integrated cost-effectiveness model. The primary source for (i) the ETAT indicators was Molyneux et al. [22], (ii) the case management of fever and malaria indicators was the evaluation of the Joint Uganda Malaria Training Program [23], (iii) the pneumonia indicators was Ayieko et al. [24], and (iv) the HIV indicators was the MOH's Quality Assurance Program [25]. For TB indicators, the primary sources were the MOH's Intensive Case Finding Form [26], the World Health Organization's treatment guidelines [27] and the Stop TB program [28], [29]. For TB and HIV indicators, the primary sources were the MOH's Quality Assurance Program [25], and United Nations' guidelines [30].

**Table 2:** Sample percentages by performance indicator, arm and time period.

Program Area, Performance Indicator, and Subgroup		Arm A		Arm B	
		Time 0	Time 1	Time 0	Time 1
**Emergency Triage, Assessment and Treatment**					
1†	Proportion of outpatients triaged				
	0–4 years	6,821 (26%)	51,337 (84%)	23,399 (39%)	42,351 (70%)
	5 or more years	18,673 (27%)	150,550 (86%)	61,644 (46%)	122,629 (73%)
2	Proportion of emergency and priority patients who were admitted, detained, or referred				
	0–4 years	334 (17%)	3,126 (39%)	2,071 (24%)	2,988 (38%)
	5 or more or more years	335 (8%)	3,357 (36%)	1,639 (11%)	2,325 (20%)
3	Estimated proportion of emergency patients who received at least one appropriate treatment				
	0–4 years	400 (45%)	1,517 (74%)	1,989 (59%)	1,728 (58%)
	5 or more years	505 (28%)	1,427 (61%)	3,110 (47%)	1,828 (49%)
**Case management of fever and malaria**					
4†	Proportion of malaria suspects with a malaria test result recorded				
	0–4 years	9,292 (42%)	26,413 (53%)	19,228 (37%)	20,971 (41%)
	5 or more years	13,947 (34%)	47,033 (50%)	25,980 (33%)	31,416 (33%)
5	Estimated proportion of malaria cases who received appropriate antimalarial treatment				
	0–4 years	9,467 (51%)	28,434 (77%)	25,002 (57%)	27,368 (66%)
	5 or more years	14,040 (42%)	44,192 (73%)	35,827 (57%)	46,520 (65%)
6†	Proportion of patients with a negative malaria test result who were prescribed an antimalarial				
	0–4 years	1,884 (56%)	4,017 (37%)	5,424 (65%)	6,334 (60%)
	5 or more years	3,528 (42%)	8,374 (27%)	7,519 (47%)	9,531 (44%)
7	Proportion of patients with a positive malaria test result who were prescribed an antibiotic				
	0–4 years	2,830 (48%)	7,714 (50%)	5,613 (52%)	5,601 (54%)
	5 or more years	2,271 (41%)	6,688 (41%)	4,149 (41%)	4,293 (44%)
**Case management of respiratory illness**					
8	Proportion of pneumonia suspects aged under 5 years assessed for pneumonia				
	0–4 years	403 (3%)	5,720 (16%)	1,732 (6%)	7,141 (21%)
9	Estimated proportion of patients aged under 5 years diagnosed with pneumonia who received appropriate antibiotic treatment				
	0–4 years	902 (56%)	2,892 (59%)	2,676 (51%)	3,351 (60%)
10†	Proportion of TB suspects with a first acid fast bacilli (AFB) smear result				
	0–13 years	32 (1%)	118 (1%)	48 (1%)	79 (3%)
	14 or more years	324 (15%)	1,359 (18%)	647 (20%)	958 (24%)
11†	Estimated proportion of patients with AFB smear negative results who received empiric treatment for acute respiratory infection				
		63 (21%)	414 (31%)	138 (24%)	255 (29%)
12	Proportion of AFB positive patients prescribed initial TB treatment or referred for TB care				
	NTLP laboratory register linked to NTLP treatment register	68 (40%)	150 (56%)	86 (61%)	91 (49%)
13†	Proportion of new TB patients with a follow-up AFB smear at 2 months				
	HIV-infected	112 (38%)	56 (31%)	138 (38%)	60 (38%)
	HIV negative	135 (36%)	92 (39%)	160 (39%)	64 (33%)
14	Proportion of new TB patients with treatment success				
	HIV-infected	124 (58%)	66 (46%)	168 (63%)	57 (46%)
	HIV negative	185 (68%)	107 (60%)	197 (66%)	82 (54%)
15	Proportion of patients in TB treatment with an HIV test result recorded				
	NTLP register	252 (90%)	477 (95%)	366 (84%)	401 (89%)
**HIV testing and prevention**					
**16** †	Proportion of patients with an HIV test result recorded				
	Revised MF5, TB suspect, 2–17 months	8 (1%)	47 (2%)	11 (1%)	24 (2%)
	Revised MF5, TB suspect,18 months to 13 years	56 (3%)	226 (4%)	95 (4%)	98 (5%)
	Revised MF5, TB suspect, 14 or more years	363 (17%)	1,730 (22%)	506 (16%)	997 (25%)
	Revised MF5, Not TB suspect, 2–17 months	162 (1%)	664 (3%)	151 (1%)	105 (0%)
	Revised MF5, Not TB suspect, 18 months to 13 years	552 (2%)	2,021 (3%)	764 (1%)	908 (1%)
	Revised MF5, Not TB suspect, 14 or more years	3,568 (7%)	14,225 (11%)	5,784 (6%)	8,524 (7%)
	ANC register, pregnant women	12,683 (67%)	29,662 (76%)	26,798 (70%)	31,924 (71%)
	ANC register, partners of pregnant women	1,578 (8%)	2,620 (7%)	5,136 (13%)	7,338 (16%)
17	Proportion of HIV-exposed infants with an HIV test result recorded				
		26 (6%)	87 (10%)	61 (8%)	92 (10%)
18	Proportion of HIV-infected pregnant women who received any ART				
		158 (90%)	368 (80%)	364 (96%)	497 (94%)
19	Proportion of HIV-infected pregnant women & infants who received ART at delivery				
	Pregnant women	59 (28%)	104 (30%)	81 (23%)	84 (20%)
	HIV-exposed infants	157 (75%)	234 (67%)	255 (71%)	299 (73%)
20	Proportion of HIV-infected pregnant women that started contraception after delivery				
		21 (75%)	25 (57%)	36 (54%)	54 (74%)
**HIV Care**					
21†	Proportion of HIV-infected patients enrolled in HIV care				
	Pregnant women	114 (27%)	418 (45%)	169 (21%)	267 (29%)
	HIV-exposed infants with a positive HIV test result	4 (44%)	17 (46%)	3 (27%)	6 (32%)
	TB patients	34 (30%)	74 (36%)	52 (28%)	39 (21%)
22†	Proportion of HIV-infected patients and HIV-exposed infants on cotrimoxazole				
	Pregnant women	285 (91%)	732 (92%)	541 (96%)	691 (93%)
	HIV-exposed infants	2 (0.47%)	6 (0.67%)	1 (0.12%)	14 (1.53%)
	TB patients	99 (100%)	168 (99%)	162 (99%)	158 (100%)
23†	Proportion of HIV-infected, ART eligible patients on life-long ART				
	Pregnant women	37 (9%)	113 (12%)	47 (6%)	69 (7%)
	HIV-exposed infants with a positive HIV test result	2 (22%)	17 (43%)	1 (9%)	5 (25%)
	TB patients	18 (16%)	45 (22%)	29 (16%)	25 (14%)

† Denotes that the indicator was a FLEI that could have been selected as the focus CQI activities.

Abbreviations: AFB = Acid-fast bacilli, ANC = Antenatal care, ART = Antiretroviral therapy, CQI = Continuous Quality Improvement, FLEI = Facility-Level Evaluation Indicator, HIV = Human Immunodeficiency Syndrome, MF5 = Medical Form 5, MOH = Ministry or Health, NTLP = National Tuberculosis and Leprosy Program, TB = Tuberculosis.

Twelve of the indicators used data from the MOH's outpatient record called the Medical Form 5 (MF5). The MF5 was initially modified by the Uganda Malaria Surveillance Project [31] and further revised by IDCAP to include information about ETAT, pneumonia, HIV testing, and drug availability. Another twelve indicators used data from nine MOH registers. The proportion of patients with an HIV test recorded (Indicator 16) relied on data from both the revised MF5 and registers. Some indicators relied on data from more than one register. For example, the proportion of Acid-Fast Bacilli (AFB) positive patients prescribed initial TB treatment or referred for TB care (Indicator 12) required linking people with an AFB smear positive result in the National TB and Leprosy Program (NTLP) laboratory register with the NTLP treatment register.

Data on two of the 25 indicators in the protocol were not available for analysis, because data on all HIV-infected patients were not collected consistently across facilities: People in HIV care with a CD4 test within the last six months (Indicator 24), and People in HIV care who were screened for TB (Indicator 25). The definitions of these indicators are in Table S1.

#### Data collection

Data were collected prospectively on every outpatient from November 2009 to December 2010 with the revised MF5, and retrospectively on every patient in the registers for the same time period. The accuracy of the data was not validated. Data from the NTLP treatment register were collected from the beginning of January 2009 for the proportion of new TB patients with a follow-up AFB smear at two months (Indicator 13), and proportion of new TB patients with treatment success (Indicator 14). In addition, missing data in the NTLP treatment register were completed by consulting with the TB focal person at each facility who had access to community-based records.

The revised MF5 forms were electronically captured with EpiInfo3.2 (United States Centers for Disease Control and Prevention, Atlanta, Georgia). From March 2010, all MF5 data entry was performed by a data entry assistant at each facility who also checked the data for completeness and worked with the health facility team to improve it. At the Arm A facilities, the data entry assistants participated in the CQI activities, but they did not systematically analyze and report results for performance indicators. Data were transmitted electronically by an internet modem or a smart phone to the Infectious Diseases Institute for cleaning and analysis. The MF5 data were merged using Microsoft Excel (Microsoft Corporation, Redmond, Washington).

The register data were extracted onto paper forms by the data entry assistant or data technicians based at the Infectious Diseases Institute and entered using Microsoft Excel. For indicators that were linked across registers, algorithms were created for matching records using Microsoft Excel and criteria were established for declaring a match. The antenatal care register is organized by visit rather than by patient, and most women had more than one visit per pregnancy. An algorithm was created for identifying women with multiple visits using Microsoft Excel and a single “de-duplicated” record was created for each woman.

### Randomization

Thirty-six facilities were randomized to two parallel arms (1∶1 balance): Arm A (IMID and OSS in Time 1, beginning in April 2010) and Arm B (IMID only in Time 1), where Time 0 was from November 2009 to March 2010 in Arm A and refers to the months before the interventions. Sites were randomized in strata to control for two other on-site interventions: 1) previous site for a national CQI program for HIV prevention and treatment, and 2) previous or current site for the Baylor International Pediatric AIDS Initiative. For more information on sequence generation, allocation, and implementation, see Naikoba et al [18], and Weaver et al. [15] The randomization was conducted on February 23, 2010 after almost four months of baseline data collection.

### Blinding

The staff, including the data entry assistants, and participants were not blinded during the interventions.

### Sample size

Sample size calculations were reported in Naikoba et al. [18] Briefly, sample sizes were calculated to test a difference between the arms with facility as the unit of analysis. The calculations were based on two facility performance indicators reported in Ssekabira et al:[23] percentage of malaria suspects with a malaria test result recorded (Indicator 4), and percentage of patients with a negative malaria test result who were prescribed an antimalarial (Indicator 6). The calculations were based on a 20% absolute difference and assumed a power of 80%, a 5% level of significance, and Gaussian distribution of indicator scores.

### Data analysis

#### Primary analyses

Descriptive statistics compared patient populations across sites. The effects of IMID training on each indicator were estimated with the pre/post change in Arm B. The combined effects of IMID training and OSS were estimated with the pre/post change in Arm A. The incremental effect of OSS was estimated with the difference in changes between Arm A and B. The time periods differed across arms, because the mid-level practitioners in Arm A attended the first two sessions of IMID training (March and April) and those in Arm B attended the final two sessions (May and June). In Arm A, baseline (Time 0) was from November 2009 to March 2010 and the intervention (Time 1) extended for nine months from April to December 2010. In Arm B, Time 0 was from November 2009 to May 2010 and Time 1 extended for seven months from June to December 2010. We also examined the time series of the percentage of each indicator by arm to verify that a linear model was appropriate.

The effects were analyzed as binomial experiments in which the unit of analysis was the facility-month for 12 indicators and the patient for 11 indicators. See Table S2 for the unit of analysis for each indicator. We used a generalized linear model for the proportion of patients managed appropriately for a given indicator with main effects for arm, time period and their interaction. In contrast to analyzing indicators as a percentage, the binomial experiments allow the precision of the estimates to vary across facilities with different numbers of patients in the analyses of facility-months. A regression model with a Poisson family and log link estimated the relative risk (RR) and ratio of relative risks (RRR). All regression analyses were clustered on the facility with robust standard errors to adjust for over-dispersion and using the Poisson instead of the binomial family. Dispersion graphs showing the range of proportions at baseline across facilities for the ETAT indicators and fever and malaria case management indicators were reported in Kinoti et al. (unpublished manuscript) and Mbonye et al. [16], respectively.

Two analyses estimated the odds ratio and ratio of odds ratios to allow for two levels of clustering. The analysis of the proportion of HIV-exposed infants with an HIV test result recorded (Indicator 17) was clustered on facility and sibling pairs. The analysis of the proportions of HIV-infected mothers and HIV-exposed infants who received ART at delivery (Indicator 18) was clustered on facility and mother-infant pairs.

Results for the interventions were presented with 99% confidence intervals (CI) and the tests were based on a 1% level of significance. A Bonferoni-type multiple comparison method would imply a 0.2% level of significance (5%/23 indicators), but we chose a less conservative level. P-values were also reported, so the results could be interpreted at other levels of significance. All analyses were performed with Stata version 11 (Statacorp, 2009 College Station, Texas).

#### Other independent variables

The 50 or more indicators in the protocol were reduced to 23 by combining them. For example, the indicators “Proportion of malaria suspects aged under five years with a malaria test recorded” and “Proportion of malaria suspects aged five or more years with a malaria test recorded” were combined into a single Indicator 4 with age as a covariate. The subgroups that were combined are listed in Table S1.

Additional independent variables were facility type, facility level, data entry assistant stationed at the site, and the strata described above. One or more independent variables for staffing were included in the analyses as appropriate and measured in quartiles: 1) the percentage of ideal clinicians assigned to the facility at baseline, where “ideal” was defined in the MOH health sector strategic plan; [19] 2) the number of clinicians who saw at least five patients during the month; 3) the percentage of ideal laboratory professionals assigned to the facility at baseline (Indicators 4, 10, 13, 15, and 16); and 4) the percentage of ideal midwives or nurse/midwives assigned to the facility at baseline (Indicators 18–20).

#### Sensitivity analyses

Several sensitivity analyses were performed, such as variance estimates with bootstrapping instead of robust standard errors. Two regression diagnostics were performed: 1) plotting of residuals, and 2) Cook's distance. Two estimates of the main model were repeated with the outliers and influential observations omitted. Two additional analyses addressed the potential co-linearity among the independent variables. Sensitivity analyses were performed with some covariates omitted from the analysis. We also estimated a linear regression model of the percentage of patients managed appropriately and calculated the variance inflation factors post-estimation.

## Results

### Participant Flow

**Figure 1:**
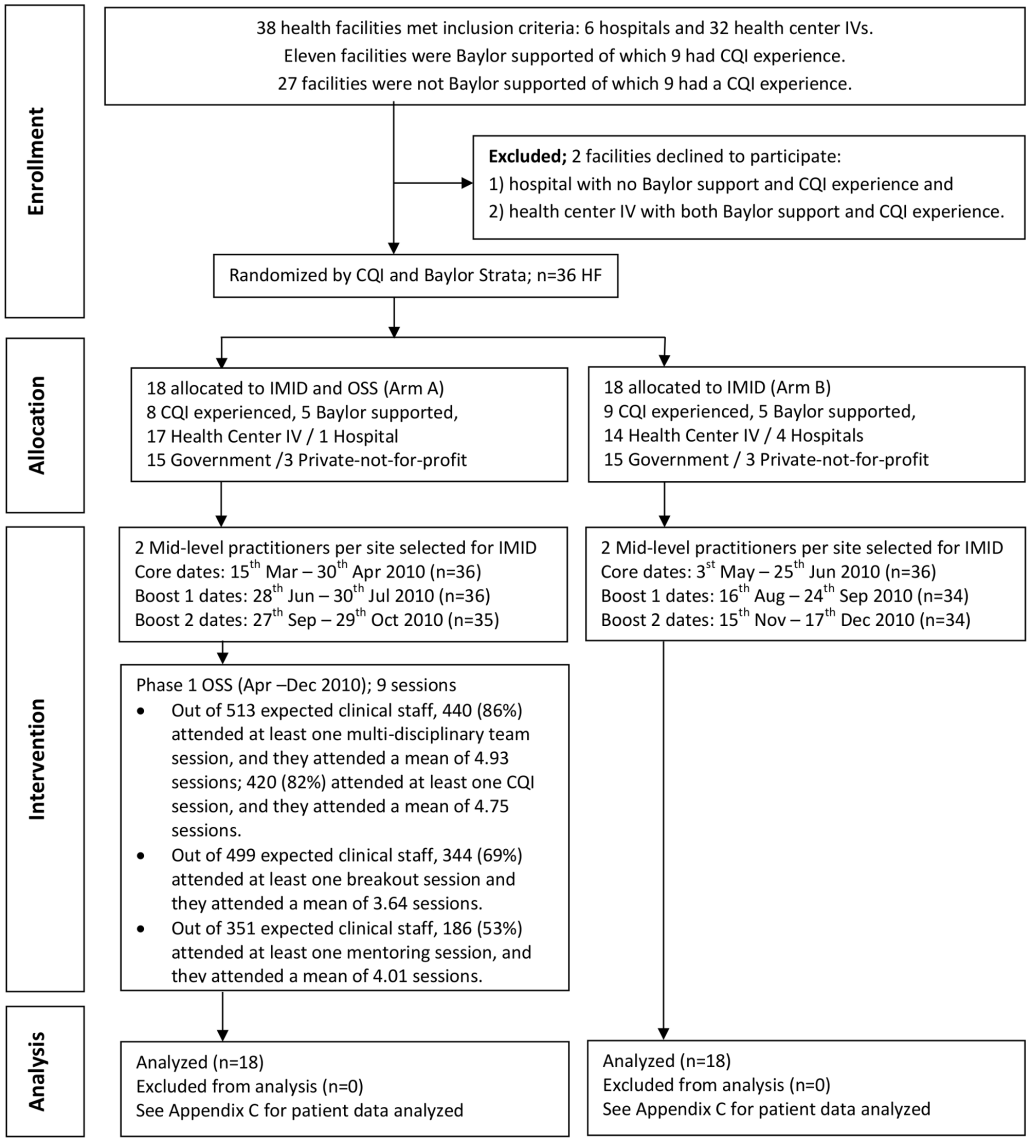
Flow diagram – recruitment and randomization. The figure shows the recruitment and randomization of health facilities, which were clusters, to two arms, and the allocation of two types of participants within each cluster: 1) mid-level practitioners who attended the Integrated Management of Infectious Disease (IMID) training, 2) clinical staff who attended on-site support (OSS) sessions. Two mid-level practitioners in arm A attended the IMID training, and clinical staff in arm A attended OSS. Two mid-level practitioners in arm B attended IMID, but clinical staff in arm B did not attend OSS. Abbreviations: Baylor support = support from the Baylor International Pediatric AIDS Initiative, CQI = Continuous Quality Improvement, IMID = Integrated Management of Infectious Disease, and OSS = On-site support.

Figure 1 reports the flow for facilities, and IMID training and OSS participants. There was no attrition among facilities. Among the 36 IMID participants in each arm, one person in Arm A and two in Arm B did not attend one or more boost courses. Consistent attendance at OSS sessions was lower. For example, only 440 out of 513 (86%) clinical staff attended at least one multi-disciplinary team training session, and average attendance was 4.93 out of nine sessions among those who attended at least one session.

Data on the number of observations used in the analysis, total number of observations, and percentage analyzed are reported in Table S2 for each indicator. The percentage analyzed for the indicators that were based on the facility-month data from the revised MF5 were generally above 95%, dropping below 95% for indicators that relied on drug stock data or were missing data on covariates for subgroups. The response rates for indicators that were based on register data were lower, especially for the proportion of HIV-infected pregnant women who started contraception after delivery (Indicator 20), which relied on the post-natal care register.

### Recruitment

The facilities were recruited between March and September 2009. For IMID participants, recruitment was between June 2009 and February 2010, and the registration and consent process were between December 2009 and March 2010. Recruitment and registration of OSS participants was ongoing, beginning in April 2010. Staff was encouraged to attend OSS sessions irrespective of previous attendance.

### Baseline data

The 36 facilities included 31 health center IV and five hospitals, 17 facilities with previous CQI experience, 10 facilities with support from the Baylor International Pediatric AIDS Initiative, and six private-not-for profit facilities. Facility descriptive statistics by arm are shown in Figure 1. Among IMID participants, 46 were clinical officers (24 in Arm A, 21 in Arm B), 22 were registered nurses (12 in Arm A and 11 in Arm B), and four were registered midwives were in Arm B.

Baseline data on each indicator in each arm and subgroup are reported in Table 2. As shown, most indicators were below 50% at baseline. In particular, four indicators for children were below 10% in both arms: 1) Pneumonia suspects assessed for pneumonia (Indicator 8), 2) TB suspects with a first AFB smear result (Indicator 10), 3) Outpatients with an HIV test recorded (Indicator 16, for both TB suspects and other outpatients), 4) HIV-exposed infants on cotrimoxazole (Indicator 22). On the opposite end of the spectrum, two indicators were above 75% in both arms: 1) Patients on TB treatment with an HIV test recorded (Indicator 15), 2) HIV-infected patients on cotrimoxazole (Indicator 22, for both pregnant women and TB patients).

### Outcomes and estimation

Table 3 reports the primary analysis for 23 indicators. Overall, IMID training was associated with statistically significant improvement in three indicators, IMID training and OSS combined was associated with improvements in six indicators, and OSS alone was associated with improvements in one indicator. As reported in Kinoti et al, (unpublished manuscript) IMID training was associated with improvements in outpatients triaged (Indicator 1: aRR = 1.29, 99%CI = 1.01,1.64) and emergency and priority patients who were admitted, detained, or referred (Indicator 2: aRR = 1.59, 99%CI = 1.04,2.44). IMID training and OSS combined were associated with improvements in all three ETAT indicators, i.e., outpatients triaged (aRR = 2.03, 99%CI = 1.13,3.64), emergency and priority patients who were admitted, detained or referred (aRR = 3.03, 99%CI = 1.40,6.56), and the estimated proportion of emergency patients who received at least one appropriate treatment (Indicator 3, aRR = 1.77, 99%CI = 1.10,2.84). The incremental effect of OSS was associated with an increase in emergency patients who received at least one appropriate treatment (aRRR = 1.84, 99%CI = 1.09,3.12). As reported in Mbonye et al. [16], IMID and OSS combined were associated with statistically significant improvements in two case management of fever and malaria indicators: the estimated proportion of malaria cases who received appropriate antimalarial treatment (Indicator 5, aRR = 1.50, 99%CI = 1.04,2.17), and patients with a negative malaria test who were prescribed an antimalarial (Indicator 6, aRR = 0.67, 99%CI = 0.46,0.97). In addition, IMID was associated with a statistically significant improvement in pneumonia suspects assessed for pneumonia (Indicator 8, aRR = 2.31, 99%CI = 1.50, 3.55). IMID and OSS combined were associated with statistically significant improvements in HIV-infected patients enrolled in HIV care (Indicator 21, aRR = 1.58, 99%CI = 1.32, 1.89).

**Table 3:** Overview of performance indicator results.

	Indicator	Combined effect of IMID training and OSS (Arm A: Time 1-Time 0		Effect of IMID training (Arm B: Time 1-Time 0)		Additional effect of OSS (Arm A vs. Arm B: Time 1-Time 0)	
		aRR (CI)	p-value	aRR (CI)	p-value	aRRR (CI)	p-value
*1* †	Proportion of outpatients triaged	2.03** (1.13, 3.64)	0.002	1.29** (1.01, 1.64)	0.007	1.58 (0.82, 3.01)	0.071
*2*	Proportion of emergency and priority patients who were admitted, detained, or referred	3.03** (1.40, 6.56)	<0.001	1.59** (1.04, 2.44)	0.005	1.91* (0.89, 4.10)	0.030
*3*	Estimated proportion of emergency patients who received at least one appropriate treatment	1.77** (1.10, 2.84)	0.002	0.96 (0.77, 1.191)	0.629	1.84** (1.09, 3.12)	0.003
*4* †	Proportion of malaria suspects with a malaria test result recorded	1.25* (0.94, 1.65)	0.045	0.97 (0.82, 1.14)	0.636	1.28* (0.93, 1.78)	0.049
*5*	Estimated proportion of malaria cases who received appropriate antimalarial treatment	1.50** (1.04, 2.17)	0.004	1.09 (0.87, 1.36)	0.315	1.38 (0.89, 2.13)	0.061
*6* †	Proportion of patients with a negative malaria test result who were prescribed an antimalarial	0.67** (0.46, 0.97)	0.006	0.96 (0.84, 1.10)	0.400	0.70* (0.48, 1.00)	0.011
*7*	Proportion of patients with a positive malaria test result who were prescribed an antibiotic	1.04 (0.88, 1.21)	0.566	1.06 (0.96, 1.17)	0.124	0.98 (0.81, 1.18)	0.747
*8*	Proportion of pneumonia suspects aged under 5 years assessed for pneumonia	2.13* (0.85, 5.32)	0.034	2.31** (1.50, 3.55)	<0.001	0.92 (0.34, 2.53)	0.835
*9*	Estimated proportion of patients aged under 5 years diagnosed with pneumonia who received appropriate antibiotic treatment	0.88 (0.64, 1.20)	0.288	1.08 (0.92, 1.27)	0.208	0.81 (0.57, 1.15)	0.127
*10* †	Proportion of TB suspects with a first acid fast bacilli (AFB) smear result	1.32 (0.78, 2.22)	0.178	1.20 (0.70, 2.05)	0.376	1.09 (0.53, 2.24)	0.745
*11* †	Estimated proportion of patients with AFB smear negative results who received empiric treatment for acute respiratory infection	1.45* (0.91, 2.31)	0.039	1.25 (0.75, 2.10)	0.263	1.16 (0.67, 1.99)	0.483
*12*	Proportion of AFB positive patients prescribed initial TB treatment or referred for TB care	1.30 (0.88, 1.92)	0.085	0.81 (0.55, 1.19)	0.154	1.61* (0.93, 2.79)	0.025
*13* †	Proportion of new TB patients with a follow-up AFB smear at 2 months	0.96 (0.73, 1.27)	0.712	0.95 (0.63, 1.42)	0.725	1.02 (0.62, 1.67)	0.934
*14*	Proportion of new TB patients with treatment success	0.87 (0.62, 1.22)	0.280	0.83 (0.61, 1.13)	0.116	1.04 (0.67, 1.63)	0.806
*15*	Proportion of TB Patients with an HIV test result recorded	1.05 (0.96, 1.14)	0.186	1.06 (0.97, 1.15)	0.089	0.99 (0.89, 1.09)	0.767
*16* †	Proportion of patients with an HIV test result recorded	1.06(0.88,1.29)	0.404	0.98 (0.82,1.16)	0.725	1.09 (0.90,1.31)	0.238
*17*	Proportion of HIV-exposed infants with an HIV test result recorded - odds ratio and ratio of odds ratios reported	1.59 (0.60, 4.21)	0.224	1.30 (0.59, 2.87)	0.396	1.22 (0.35, 4.31)	0.683
*18*	Proportion of HIV-infected pregnant women who received any ART	0.90* (0.78,1.02)	0.032	0.96 (0.90,1.03)	0.115	0.93 (0.79,1.10)	0.250
*19*	Proportion of HIV-infected pregnant women and infants who received ART at delivery – odds ratio and ratio of odds ratios reported	0.90 (0.70, 1.14)	0.238	0.95 (0.78, 1.17)	0.542	0.94 (0.70, 1.28)	0.616
*20*	Proportion of HIV-infected pregnant women that started contraception after delivery	0.63 (0.21, 1.93)	0.288	0.86 (0.49, 1.53)	0.505	0.73 (0.22, 2.45)	0.506
*21* †	Proportion of HIV-infected patients enrolled in HIV care	1.58** (1.32, 1.89)	<0.001	1.23 (0.85, 1.77)	0.145	1.28 (0.86, 1.91)	0.106
*22* †	Proportion of HIV-infected patients and HIV-exposed patients on cotrimoxazole	1.00 (0.90, 1.12)	0.973	0.99 (0.94, 1.04)	0.534	1.01 (0.90, 1.15)	0.774
*23* †	Proportion of HIV-infected, ART eligible patients on lifelong ART	1.42* (0.91,2.22)	0.042	1.13 (0.77, 1.67)	0.414	1.25 (0.68, 2.30)	0.336

† Denotes that the indicator was a FLEI that could have been selected as the focus CQI activities.

** Denotes that the effect of the intervention was significant at the .01 level.

* Denotes that the effect of the intervention was significant at the .05 level. The 99% confidence intervals (CI) are based on the .01 level of significance.

Abbreviations: aRR = adjusted relative risk, aRRR = adjusted ratio of relative risks, AFB = Acid-fast bacilli, ART = Antiretroviral therapy, CI = confidence interval, CQI = Continuous Quality Improvement, FLEI = Facility-Level Evaluation Indicator, HIV = Human Immunodeficiency Syndrome, IMID = Integrated Management of Infectious Disease, OSS = On-site support, RR = relative risk, RRR = ratio of relative risks, TB = Tuberculosis.

Table 4 shows the number of Arm A facilities that selected each FLEI. Three of the widely adopted indicators were associated with statistically significant combined effects of IMID and OSS: outpatients triaged (Indicator 1), patients with a negative malaria test result who were prescribed an antimalarial (Indicator 6), and HIV-infected pregnant women enrolled in HIV care (Indicator 21). FLEI that were adopted by fewer than half the sites were generally not associated with statistically significant changes.

**Table 4:** Exploratory analysis: Comparison of Arm A that adopted a Facility-Level Evaluation Indicators (FLEI) to those that did not.

			Combined effect of IMID training & OSS (Time 1-Time 0)						Effect of OSS: Arm A vs. Arm B (Time 1 – Time 0)					
Indicator	Performance Indicator	Number with FLEI	(Arm A: Table 2)		Arm A FLEI		Arm A no FLEI		(Arm A vs. Arm B: Table 2)		Arm A FLEI vs. Arm B		Arm A no FLEI vs. Arm B	
			aRR (CI)	p-value	aRR (CI)	p-value	aRR (CI)	p-value	aRRR (CI)	p-value	aRRR (CI)	p-value	aRRR (CI)	p-value
1	Proportion of outpatients triaged	16	2.03**	0.002	2.03**	0.007	1.99**	0.008	1.58	0.071	1.58	0.111	1.55	0.115
			(1.13,		(1.03,		(1.02,		(0.82,		(0.75,		(0.76,	
			3.64		4.03)		3.88)		3.01)		3.31)		3.15)	
4	Proportion of malaria suspects for whom a blood smear was ordered and test results recorded	15	1.25*	0.045	1.31*	0.029	1.01	0.942	1.28*	0.049	1.36*	0.027	1.04	0.747
			(0.94,		(0.95,		(0.74,		(0.93,		(0.95,		(0.74,	
			1.65)		1.82)		1.37)		1.78)		1.96)		1.48)	
6	Proportion of patients with a negative malaria test who were prescribed an antimalarial	13	0.67**	0.006	0.64*	0.024	0.71**	0.003	0.70*	0.011	0.67*	0.038	0.74*	0.013
			(0.46,		(0.38,		(0.53,		(0.48,		(0.40,		(0.55,	
			0.97)		1.07)		0.95)		1.00)		1.10)		1.01)	
10	Proportion of TB suspects with a first acid fast bacilli (AFB) smear result	10	1.32	0.178	1.65*	0.027	0.89	0.713	1.09	0.745	1.33	0.388	0.72	0.388
			(0.78,		(0.92,		(0.38,		(0.53,		(0.57,		(0.26,	
			2.22)		2.95)		2.05)		2.24)		3.11)		1.94)	
11	Estimated proportion of patients with AFB smear negative results prescribed an empiric treatment for acute respiratory infection and drug in stock	0	1.45*	0.039		1.16	0.483							
			(0.91,			(0.67,								
			2.31)			1.99)								
13	Proportion of new TB patients with a follow-up AFB smear at 2 months	2	0.96	0.723	0.93	0.657	0.98	0.900	1.02	0.927	0.98	0.948	1.04	0.837
			(0.73,		(0.61,		(0.71,		(0.62,		(0.55,		(0.62,	
			1.27)		1.42)		1.37)		1.67)		1.77)		1.76)	
16	Proportion of patients who have HIV test results recorded		1.06	0.404					1.09	0.238				
			(0.88,						(0.90,					
			1.29)						1.31)					
	Outpatients	5	1.50*	0.019	1.19	0.458	1.83	0.018	1.37	0.128	1.10	0.730	1.68	0.066
			(0.96,		(0.64,		(0.95,		(0.80,		(0.55,		(0.81,	
			2.35)		2.21)		3.55)		2.38)		2.19)		3.50)	
	TB suspects	8	1.07	0.686	1.07	0.693	1.06	0.841	0.92	0.731	0.92	0.729	0.91	0.802
			(0.69,		(0.67,		(0.48,		(0.48,		(0.50,		(0.35,	
			1.66)		1.72)		2.34)		1.75)		1.70)		2.37)	
21	Proportion of HIV-infected patients enrolled in HIV care		1.58**	<0.001					1.28	0.106				
			(1.32,						(0.86,					
			1.89)						1.91)					
	Pregnant women	10	1.69**	<0.001	1.87**	<0.001	1.40*	0.040	1.25	0.206	1.37	0.071	1.03	0.894
			(1.35,		(1.47,		(0.92,		(0.79,		(0.87,		(0.57,	
			2.11		2.38)		2.14)		1.96)		2.17)		1.85)	
22	Proportion of HIV-infected patients and HIV-exposed infants on cotrimoxazole		1.00	0.973					1.01	0.774				
			(0.90,						(0.90,					
			1.12)						1.15)					
	Pregnant women	6	1.00	0.988	0.92*	0.041	1.06	0.545	1.04	0.537	0.95	0.334	1.10	0.335
			(0.86,		(0.82,		(0.83,		(0.88,		(0.84,		(0.85,	
			1.16)		1.02)		1.35)		1.23)		1.08)		1.42)	
	TB Patients	6	0.99	0.359	1.00	0.421	0.99	0.307	0.98	0.191	0.99	0.348	0.98	0.170
			(0.98,		(0.99,		(0.97,		(0.94,		(0.96,		(0.94,	
			1.01)		1.01)		1.02)		1.02)		1.02)		1.02)	
23	Proportion of HIV-infected, ART eligible patients who are on lifelong ART		1.42*	0.042					1.25	0.336				
			(0.91,						(0.68,					
			2.22)						2.30)					
	Pregnant women, All on ART FLEI	10	1.39	0.083	1.28	0.364	1.65*	0.014	1.16	0.575	1.07	0.838	1.37	0.246
			(0.85,		(0.63,		(0.97,		(0.59,		(0.46,		(0.68,	
			2.28)		2.60)		2.79)		2.27)		2.46)		2.78)	
	TB patients, All on ART FLEI	10	1.35	0.183	1.02	0.939	1.99**	0.006	1.66	0.150	1.27	0.575	2.47*	0.011
			(0.75,		(0.44,		(1.04,		(0.67,		(0.42,		(0.98,	
			2.43)		2.39)		3.81)		4.10)		3.81)		6.19)	

** Denotes that the effect of the intervention was significant at the .01 level.

* Denotes that the effect of the intervention was significant at the .05 level. The 99% confidence intervals (CI) are based on the .01 level of significance.

Abbreviations: aRR = adjusted relative risk, aRRR = adjusted ratio of relative risks, AFB = Acid-fast bacilli, ART = Antiretroviral therapy, CI = confidence interval, CQI = Continuous Quality Improvement. FLEI = Facility-Level Evaluation Indicator, HIV = Human Immunodeficiency Syndrome, IMID = Integrated Management of Infectious Disease, OSS = On-site support, RR = relative risk, RRR = ratio of relative risks, TB = Tuberculosis.

#### Sensitivity analyses

The data entry assistant arrived at the sites in March 2010, which left only one month of baseline data in Arm A and three months in Arm B during which they were at the sites. The estimates were repeated without the data entry assistant as a covariate, which, in general, increased the size of the main effects and decreased their standard errors. There was no other evidence of multiple co-linearity in the estimates, such as large changes in coefficients when covariates were removed. Further, the variance inflation factors were calculated for several indicators that were based on different data sources (Indicators 1, 4, 8, 10, 16, 17, 18 and 21), and they were not larger than four for any independent variable in any estimates. Other results of the sensitivity analyses for ETAT and case management of fever and malaria indicators are reported in Kinoti et al. (unpublished manuscript) and Mbonye et al. [16], respectively.

#### Ancillary Analyses

The performance indicators that were “Facility-Level Evaluation Indicators” (FLEI) are noted by a dagger (†) next to the indicator number in Tables 2 and 3. To explore the effects of selecting a FLEI as part of the CQI activities, an analysis was conducted with three arms: Arm A sites that adopted the FLEI associated with the indicator (“Arm A FLEI”), Arm A sites that did not adopt the FLEI (“Arm A no FLEI”), and Arm B. The results, reported in Table 4, showed larger adjusted relative risks (RR) and ratios of RR (aRRR) for Arm A sites that adopted the FLEI than for Arm A sites that did not for most indicators, suggesting that the “Arm A no FLEI” sites may have diluted the effect of the CQI activities in the primary analysis of Arm A. Two notable exceptions where the “Arm A no FLEI” effects were larger than the “Arm A FLEI” effects were: patients with an HIV test result recorded (Indicator 16 for the outpatient subgroup); and HIV-infected, ART eligible patients on lifelong ART (Indicator 23, for pregnant women and TB patients).

## Discussion

The overview of results on 23 facility performance indicators in this article reported that IMID was associated with statistically significant increases in three indicators in Arm B: outpatients triaged (Indicator 1), emergency and priority patients who were admitted, detained, or referred (Indicator 2), and pneumonia suspect aged under five years assessed for pneumonia (Indicator 8). The combination of IMID and OSS was associated with statistically significant increases in six indicators in Arm A: Indicators 1 and 2 above plus emergency patients who received at least one appropriate treatment (Indicator 3), malaria cases who received appropriate treatment (Indicator 4), patients with a negative malaria test result who were prescribed an antimalarial (Indicator 6), HIV-infected patients enrolled in HIV care (Indicator 20). The additional effect of OSS was statistically significant for Indicator 3.

Two characteristics distinguished the six indicators for which the combined effects of IMID and OSS were statistically significant from the other indicators. Five of the six indicators were associated with the first two OSS sessions, which were “Emergency Care in Adults and Children,” and “Fever and Malaria Case Management,” suggesting that either the novelty of the interventions or more time to work on improvements contributed to success. Three of the six indicators were FLEI that were adopted by 10 or more health facilities, suggesting that the CQI process, in which staff selected and focused on particular indicators, contributed to success. (The other three of the six indicators were not FLEI.) The ancillary analysis in Table 4 showed that the effect sizes were usually larger among Arm A sites that adopted a particular FLEI than among Arm A sites that did not. Admittedly, the sample size of the trial was not powered for this secondary analysis. These characteristics suggest that improvements among the teams at the health facilities may take several months and a concentrated effort.

As one of the first randomized trials of an integrated educational outreach and quality improvement intervention, the absence of an incremental effect of OSS on the majority of indicators should not detract from the impressive success on ETAT (Kinoti et al. unpublished manuscript) and fever and malaria case management indicators [16]. In light of the successful Joint Uganda Malaria Training Program [23] results, in which two of the four performance indicators showed statistically significant improvements, IDCAP may have aimed too high. In tripling the length of the training program from a one-week course and three follow-up visits to a three-week course and nine follow-up visits, it may have been more reasonable to hypothesize that IDCAP would triple the effects of the Joint Uganda Malaria Training Program, i.e., statisitically significant improvements in six performance indicators, which was accomplished.

Alternatively, improvements in 23 performance indicators across multiple diseases and program areas may have required more OSS sessions over a longer time period. A systematic review of quality improvement collaboratives identified only two randomized controlled trials, and reported that the impact was statistically significant for only the longer term collaborative with ongoing data collection and communication [32]. If educational outreach and CQI activities were to continue for two or more years, the intervention would need a process in which facilities that achieved their initial CQI goals would then select additional indicators.

Attendance at OSS sessions was lower than the IMID training at the Infectious Diseases Institute. Lower attendance at the on-site sessions could be explained by staff who were absent to treat emergency patients at a functioning clinic, and staff assigned to evening and night shifts who were off-duty during the sessions, as well as absenteeism among health professionals in general [33]. The evidence showed that it would be difficult to reach all staff during a single visit. The evidence also raised the question of whether or not processes adopted by the participants were shared with staff who missed a session.

The IDCAP trial was a valuable experience in defining and collecting integrated facility performance indicators, and provided excellent baseline data for subsequent research on TB at some of the facilities [Yuka Manabe, personal communication], and clinical mentoring at others [Sarah Naikoba, personal communication]. In addition, the paths of the performance indicators over time could potentially be analyzed to generate hypotheses about the timing and duration of the effects for future research. Finally, the data entry assistant contributed to statistically significant improvements to two of the 12 indicators that were based on data from the revised MF5 form. During IDCAP, the data collection and analysis were separate from the interventions. A systematic review of teaching quality improvement to clinicians suggested that the impact of the data entry assistant on quality of care could be greater if they analyzed the data to feedback to the CQI process [34].

### Limitations

There were several limitations to the research design. The pre/post component did not control for other changes at the sites over the course of 14 months. The 36 sites were located in 28 different districts however, so an initiative at the district-level generally would have affected only one site. The cluster randomized trial component measured the effect of OSS in addition to IMID. To the extent that there was an interaction between IMID training and OSS, the effect of OSS would not be the same in the absence of the IMID training. Finally, the analysis assumed that the IMID training was identical in both arms, but the participants in Arm B attended later sessions than in Arm A. Although the IMID curriculum and faculty were identical in both arms, the quality of IMID training may have improved with practice, as evidenced by the larger effect of the IMID training on Pneumonia suspects aged under 5 years assessed for pneumonia (Indicator 8) in Arm B than Arm A. It was not possible to train everyone at once, because the Infectious Diseases Institute sought to limit the courses to 25 or fewer participants per session. Participants in Arm A attended sessions first, to minimize the delay between the IMID core course and OSS.

These facility performance results must be considered in the context of the effects of the IDCAP interventions on clinical competence [15] and practice, and under-five mortality, all of which were measured as part of IDCAP. With regard to sample size, the evaluation was designed to detect a difference between arms at a 5% level of significance. The statistical tests however, tested a pre/post difference within arms and a difference in ratios between arms at a 1% level of significance. The effect of OSS was measured by the interaction of time and arm, where interaction effects generally have less power and larger standard errors than simply detecting a difference between arms. Although the sample size may have been too small to detect the effects of OSS at the 1% level of significance, the results will provide researchers with the information they need to calculate sample sizes for future trials.

Finally, IDCAP focused on capacity-building interventions at health facilities rather than the larger health system or community-based interventions. Integrated interventions, such as Integrated Management of Childhood Illness [6], [7], [10] included interventions for the health system, health facilities, and community. IDCAP sought to test the pure effects of capacity building at health facilities, but we readily acknowledge the essential role of health systems [35] and community-based interventions in improving health outcomes.

### Generalizability

The generalizability of the IDCAP results was limited, because the eligibility criteria focused on a narrow range of health facilities to isolate the effect of OSS. Strictly speaking, the results would only generalize to Ugandan health center IV that met the inclusion criteria. To the extent that similarities exist among primary care settings in sub-Saharan Africa, the results would inform programs that serve populations at risk for HIV, malaria, pneumonia, TB, and other common infectious diseases.

The effect of OSS was statistically significant at the 1% level for only one of 23 facility performance indicators, meaning that the results for the incremental effect of OSS would generalize for only that indicator. The p-value was less than 0.05 for four additional indicators showing some, albeit weak, evidence of effectiveness: 1) emergency and priority patients who were admitted, detained or referred (Indicator 2), 2) malaria suspects with a malaria test result recorded (Indicator 4), 3) patients with a negative malaria test who were prescribed an anti-malarial (Indicator 6), and 4) AFB positive patients prescribed initial TB treatment or referred for TB care (Indicator 12). Given the limitations of the sample size calculations, the IDCAP results were encouraging, but were not definitive evidence of the incremental effect of OSS.

## Conclusions

Many interventions are designed for a single disease or program area. The IDCAP baseline results however, showed that there was the potential to improve the quality of care across multiple infectious diseases and program areas. In many primary care facilities improving care would require a multi-step process for a team of professionals, rather than training a single person at a single point in time.

A combination of on-going training and quality improvement interventions were associated with statistically significant improvements, primarily in performance indicators for ETAT, malaria, pneumonia and enrollment in HIV care. The trial results however, showed that the OSS intervention significantly improved performance in only one of 23 facility indicators. Companion articles have or will address the effects on other measures, and the cost-effectiveness of the IDCAP interventions.

## Supporting Information

Table S1 **Definitions and data sources for facility performance indicators.**
(DOCX)

Table S2 **Total sample size and response rate for facility performance indicators by arm and time period.**
(DOCX)

Checklist S1 **CONSORT checklist for cluster randomised trials.**
(DOCX)

Protocol S1 **Integrated Infectious Disease Capacity-Building Evaluation (IDCAP).** This is the final protocol approved by the Makerere University School of Medicine Research and Ethics Committee, which was submitted as the sixth modification on November 2011.
(DOC)
