# Correction: Improving Facility Performance in Infectious Disease Care in Uganda: A Mixed Design Study with Pre/Post and Cluster Randomized Trial Components

**DOI:** 10.1371/journal.pone.0148672

**Published:** 2016-01-29

**Authors:** Marcia R. Weaver, Sarah M. Burnett, Ian Crozier, Stephen N. Kinoti, Ibrahim Kirunda, Martin K. Mbonye, Sarah Naikoba, Allan Ronald, Timothy Rubashembusya, Stella Zawedde, Kelly S. Willis

One of the 23 facility performance indicators has been redefined since the publication of this article.

The published estimates of the “Estimated proportion of emergency patients who received at least one appropriate treatment” included laboratory tests, and being admitted, detained or referred, as well as drug treatment. Although the published estimates were correct, in the analysis of the follow-up data, we redefined the indicator to eliminate the overlap with a second indicator “Proportion of emergency and priority patients who were admitted, detained, or referred,” and focused only on drug treatments.

The revised crude percentages in arm A were 35% in time 0 and 63% in time 1 among children 0–4 years and 20% in time 0 and 48% in time 1 among patients 5 and above.

In arm B they were 41% in time 0 and 48% in time 1 among children 0–4 years and 27% in time 0 and 37% in time 1 among patients 5 and above.

The revised results of the analysis were that the estimated proportion of emergency patients that received at least one appropriate treatment increased in arm A (aRR = 1.75; 99%CI: 0.99, 3.06, and was unchanged in arm B, (aRR = 1.04; 99%CI: 0.80, 1.35; aRRR = 1.68; 99%CI: 0.94, 3.00).

The revised conclusion is that the trial showed that the OSS intervention did not statistically significantly improve facility performance for any of the 23 indicators.
