# Supplementary material for: Improving Facility Performance in Infectious Disease Care in Uganda: A Mixed Design Study with Pre/Post and Cluster Randomized Trial Components
Source: PLoS One. 2014 Aug 18;9(8):e103017. doi: 10.1371/journal.pone.0103017 (PMC4136733; doi:10.1371/journal.pone.0103017)
Supplement: Table S1 — Definitions and data sources for facility performance indicators. (DOCX) [file pone.0103017.s001.docx]

**Table S1. Definitions and data sources for facility performance indicators**

|  | **Program Area and Perform-ance Indicator** | **Definition** | **Sub-groups** | **Data Source** | **Refer-ence** |
| --- | --- | --- | --- | --- | --- |
| **Emergency Triage, Assessment and Treatment (ETAT)** | | |  |  |  |
| 1† | Proportion of outpatients triaged | **Numerator:** Number of outpatients triaged, meaning that the patient was classified as emergency, priority, or queue, or an emergency sign was noted in the triage section of the form. **Denominator:** Number of outpatients | 0-4 years, 5 or more years | Revised Medical Form 5 | [22] |
| 2 | Proportion of emergency and priority patients who were admitted, detained or referred | **Numerator:** Number of emergency and priority patients admitted, detained or referred for care. **Denominator:** Number of outpatients classified as emergency or priority or an emergency sign was noted in the triage section of the form | 0-4 years, 5 or more years | Revised Medical Form 5 | [22] |
| 3 | Estimated proportion of emergency patients who received at least one appropriate treatment | **Numerator:** Number of emergency patients who received at least one treatment prescribed according to ETAT standards where the standards were reported in Kinoti et al. (unpublished manuscript). For emergency patients who were prescribed treatment and data on drug availability were missing, we applied the “in-stock” rate for patients with those data. **Denominator:** Number of outpatients classified as emergency or an emergency sign was noted in the triage section of the form | 0-4 years, 5 or more years | Revised Medical Form 5 |  |
| **Case management of fever and malaria** | | |  |  |  |
| 4† | Proportion of malaria suspects with a malaria test result recorded | **Numerator:** Number of malaria suspects with a result for a laboratory test or rapid diagnostic test for malaria, where the definition of a malaria suspect was reported in Mbonye et al. [16]**. Denominator:** Number of malaria suspects | 0-4 years, 5 or more years | Revised Medical Form 5 | [23] |
| 5 | Estimated proportion of malaria cases who received an appropriate antimalarial | **Numerator**: Number of outpatients treated with appropriate anti-malarial(s), where appropriate antimalarial treatments were quinine and four artemisinin-based combination therapies reported in Mbonye et al. [16]. For patients who were prescribed an antimalarial and data on drug availability were missing, we applied the “in-stock” rate for patients with those data. **Denominator**: Number of outpatients treated for malaria | 0-4 years, 5 or more years | Revised Medical Form 5 | [23] |
| 6† | Proportion of patients with a negative malaria test result who were prescribed an antimalarial | **Numerator:** Number of patients with a negative malaria test result prescribed any antimalarial including appropriate treatments and those that do not comply with Ugandan national guidelines. **Denominator:** Number of patients with a negative malaria test result | 0-4 years, 5+ years | Revised Medical Form 5 | [23] |
| 7 | Proportion of patients with a positive malaria test result who were prescribed an antibiotic | **Numerator**: Number of patients with a positive malaria test result prescribed any antibiotic(s), where antibiotic treatment refers to the 31 drugs listed in Mbonye et al. [16]. **Denominator:** Number of patients with a positive malaria test result | 0-4 years, 5 or more years | Revised Medical Form 5 |  |
| **Case management of respiratory illness** | | |  |  |  |
| 8 | Proportion of pneumonia suspects aged under 5 years assessed for pneumonia | **Numerator:** Number of child pneumonia suspects with at least one of the three following assessment results recorded: 1) abnormal chest sounds, 2) chest in-drawing, and 3) rapid breaths per minute. A pneumonia suspect was defined as any child aged under five years presenting with cough or who received a diagnosis of “pneumonia” or “cough/cold no pneumonia”**. Denominator:** Number of child pneumonia suspects**. Note:** The definition of suspect focused on children with cough; difficulty in breathing was inadvertently omitted from the form. |  | Revised Medical Form 5 | [24] |
| 9 | Estimated proportion of patients aged under 5 years diagnosed with pneumonia who received appropriate antibiotic treatment | **Numerator:** Number of children diagnosed with pneumonia treated with appropriate antibiotic, where appropriate antibiotic treatment referred to six drugs on the revised Medical Form 5: amoxicillin, benzyl penicillin, erythromycin, chloramphenicol, gentamicin, cotrimoxazole, and 11 other drugs that were specified: ampicillin, azithromycin, cefixime, ceftriaxone, cefuroxime, co-amoxiclav, gatifloxacin, levofloxacin, penicillin, phenoxymethylpenicillin, ampiclox (amoxicillin and cloxacillin). For patients who were prescribed an antibiotic and data on drug availability were missing, we applied the “in-stock” rate for patients with those data. **Denominator:** Number of children diagnosed with pneumonia |  | Revised Medical Form 5 |  |
| 10† | Proportion of TB suspects with a first Acid-Fast Bacilli (AFB) smear result | **Numerator**: Number of TB suspects who get a first AFB smear result, where TB suspect was defined as anyone with a history of: cough for longer than two weeks, cough for less than two weeks and night sweats, cough for less than two weeks and weight loss, TB test ordered, new TB diagnosis, started on initial TB treatment, or referred for TB treatment. For children, the definition extended to anyone who had contact with someone with TB. **Denominator**: Number of TB suspects. **Note:** The definition of TB suspect is from the Intensified Case Finding Form for People Living with HIV, contacts of smear positive patients, and HIV care settings.[27] Increasing AFB smears among TB suspects will increase case detection, but this is not the case detection indicator used by the Stop TB program.[29] | 0-13 years, 14 or more years | Revised Medical Form 5 |  |
| 11† | Estimated proportion of patients with AFB smear negative results who received empiric treat-ment for acute respiratory infection | **Numerator:** Number of people with AFB smear negative test results who received empiric treatment for acute respiratory infection, including amoxicillin, doxycycline, or erythromycin. For patients who were prescribed an antibiotic and data on drug availability were missing, we applied the “in-stock” rate for patients with those data. **Denominator:** Number of people with AFB smear negative result |  | Revised Medical Form 5 |  |
| 12 | Proportion of AFB positive patients prescribed initial TB treatment or referred for TB care | **Numerator:** Number of patients with AFB smear positive results in the NTLP laboratory register who were also listed in NTLP treatment register**. Denominator:** Number of patients with AFB smear positive results in the NTLP laboratory register |  | NTLP laboratory register linked to NTLP Treatment register | Indica-tor 11 [25] (HIV only) |
| 13† | Proportion of new TB patients with a follow-up AFB smear at 2 months | **Numerator**: Number of new TB patients in the NTLP treatment register who had follow-up AFB smear at 2 months. **Denominator**: Number of new TB patients in the NTLP treatment register. **Note:** New TB patient means that they were not classified as a re-treatment case based on their regimen. | HIV status and TB dis-ease class | NTLP treatment register | [27] |
| 14 | Proportion of new TB patients with treatment success | **Numerator:** Number of smear-positive patients on TB treatment who were cured or completed treatment and smear-negative patients on TB treatment who complete treatment. **Denominator:** Number of new TB patients in NTLP treatment register net of patients who transferred out. **Note:** The original indicators distinguished TB cure among AFB smear positive patients from TB treatment completion for other patients. These indicators were combined with a focus on completion in the analysis of TB success. | HIV status and TB dis-ease class | NTLP register | [28] |
| 15 | Proportion of patients in TB treatment with an HIV test result recorded | **Numerator:** Number of patients in TB treatment with an HIV test result recorded. **Denominator:** Number of TB patients in NTLP treatment register. **Note**: This indicator was analyzed separately from Indicator 16 below, because of the overlap between TB patients and TB suspects. |  | NTLP treatment register | Indicator C.1.1 [29] |
| **HIV testing and prevention** | | |  |  |  |
| 16† | Proportion of patients with an HIV test result recorded | **Numerator:** Number of outpatients who were not TB suspects with an HIV test result recorded. TB suspect is defined for Indicator 10. **Denominator:** Number of outpatients who were not TB suspects**. Note:** This indicator included anyone who said they knew their HIV status in the protocol, and was revised to comply with the MOH definition based strictly on a laboratory test result on the day of the outpatient visit. | 2-17 month, 17 month to 13 years, 14 or more years | Revised Medical Form 5 | Indicaor 7 [30] |
|  |  | **Numerator:** Number of TB suspects with an HIV test result recorded. TB suspect is defined for Indicator 10. **Denominator:** Number of TB suspects**. Note:** This indicator included anyone who said they knew their HIV status in the protocol, and was revised as described above. | 2-month to 13 years, 14 or more years | Revised Medical Form 5 |  |
|  |  | **Numerator:** Number of pregnant women who attended an ANC visit in a given month who had an HIV test that month with HIV status recorded. **Denominator**: Number of pregnant women who attended an ANC visit in a given month. **Note:** The numerator only includes women who had an HIV test that month and excludes those who may have had an HIV test results from a previous visit. | 14 or more years | Summary data from Health Management Information System form 105, cross-checked in ANC register by data entry assistant |  |
|  |  | **Numerator:** Number of partners of pregnant women who attended who attended an ANC visit in a given month who had an HIV test that month. **Denominator**: Number of pregnant women who attend at least one ANC visit. **NOTE:** The ANC register has space for one partner only. | 14 or more years | Summary data from Health Management Information System form 105, cross-checked in ANC register by data entry assistant |  |
|  |  | **Numerator:** Number of HIV-exposed infants aged over 18 months whose mothers attended at least one post-natal care visit who have confirmed HIV status. **Denominator:** Number of HIV-exposed infants whose mothers attended at least one post-natal care visit. **Note:** This indicator was not included in the analysis, because most children did not return to the facility for post-natal care visits 18 months after birth, and 18 month follow-up exceeded the duration of data collection after the interventions began. |  | Post-natal care register | Indicator 27a [25] |
| 17 | Proportion of HIV-exposed infants with an HIV test result recorded | **Numerator:** Number of HIV-exposed infants whose mothers attended at least one ANC visit with DNA PCR test result recorded. **Denominator:** Number of HIV-infected women who attended at least one ANC visit and whose estimated date of delivery was before July 2011. |  | ANC register, linked to Early Infant Diagnosis laboratory register | Indicator 27a [25] |
| 18 | Proportion of HIV-infected pregnant women who received any ART | **Numerator:** Number of HIV-infected pregnant women who were prescribed a drug regimen to prevent maternal to child transmission of HIV or any three-drug ART combination in ANC or ART register. **Denominator:** Number of HIV-infected pregnant women who attend at least one ANC visit |  | ANC register linked to ART registers |  |
| 19 | Proportion of HIV-infected pregnant women and their infants who received ART at delivery | **Numerator:** Number of HIV-infected pregnant women who received ART at deliver at facility, including lamivudine/nevirapine (3TC/NVP) or any three-drug combination. **Denominator:** Number of HIV-infected pregnant women who delivered at facility. **Note:** Denominator originally was all women with at least one ANC visit who were HIV-infected. The revised definition used in the analysis reflects only a subset of people who received a drug regimen to prevent maternal to child transmission of HIV. |  | Maternity register | Indicator 5 [30] |
|  |  | **Numerator before January 2011:** Number of HIV-exposed newborns who were given nevirapine and zidovudine (NVP and AZT) or nevirapine only after delivery at facility. **Denominator**: Number of HIV-exposed children delivered at facility. Note that PMTCT guidelines to prevent maternal to child transmission of HIV changed in October 2010 to nevirapine only. |  | Maternity register |  |
| 20 | Proportion of HIV-infected pregnant women that started contraception after delivery | **Numerator:** Number of HIV-infected mothers who started contraception. Contraceptive methods included oral contraceptives, progestogen-only pills, Depo Provera, Norplant, intra-uterine devices, condoms, natural family planning, abstinence, bi-tubal ligation, and others as coded in the MOH register. **Denominator:** Number of HIV-infected mothers who attended at least one post-natal care visit. **Note:** The denominator was HIV-infected women who attended at least one ANC visit in the protocol, but we did not link the ANC and post-natal care register data. In addition, contraception was to start within six weeks of delivery, but post-natal care register did not record date that contraception was prescribed. |  | Post-natal care register | Indicator 3b [25] |
| **HIV Care** | | |  |  |  |
| 21† | Proportion of HIV-infected patients enrolled in HIV care | **Numerator:** Number of HIV-infected pregnant women who attended at least one ANC visit who were registered in the Pre ART or ART register. **Denominator:** Number of HIV-infected pregnant women who attended at least one ANC visit |  | ANC register linked to Pre-ART, and ART registers | Indicator 10 [25] |
|  |  | **Numerator:** Number of infants with a positive DNA PCR HIV test result who were registered in the Pre-ART or ART Register. **Denominator:** Number of infants with a positive DNA PCR test result. **Note:** The MOH indicator included all HIV-exposed children, whereas this analysis focused on children who were HIV-infected. |  | Post-natal care register and Early Infant Diagnosis laboratory register linked to Pre-ART or ART Register | Indicator 30 [25] |
|  |  | **Numerator:** Number of TB-HIV co-infected patients who were registered in the Pre-ART or ART register**. Denominator:** Number of patients in TB treatment who were HIV-infected |  | NTLP register linked to Pre-ART, and ART registers | Indicator C.4.1 [29] |
|  |  | **Numerator:** All other HIV-infected patients enrolled in HIV care. **Denominator:** All other HIV-infected patients. **Note:** These data were not available for the analysis. |  |  |  |
| 22† | Proportion of HIV-infected patients and HIV-exposed infants on cotrimoxazole | **Numerator:** Number of HIV-infected pregnant women who attended at least one ANC visit and were prescribed cotrimoxazole prophylaxis in ANC, Pre-ART or ART register. **Denominator:** Number of HIV-infected pregnant women who attended at least one ANC visit. **Note:** HIV-infected pregnant women were added to the analysis to consistently report on the same subgroups across the three HIV care indicators. |  | ANC register linked to Pre-ART, and ART registers |  |
|  |  | **Numerator:** Number of HIV-exposed infants whose mothers attended at least one ANC visit and post-natal care register records indicated that they were prescribed cotrimoxazole prophylaxis**. Denominator:** Number of HIV-infected women who attended at least one ANC visit**. Note:** The original definition excluded children with a negative HIV test result, but they were only 3 of 607 children included in the analysis. |  | ANC register linked to post-natal care register | Indicator 8b1 [25] |
|  |  | **Numerator:** Number of TB-HIV co-infected patients who were prescribed cotrimoxazole prophylaxis in NTLP treatment, Pre-ART or ART register. **Denominator:** Number of patients in TB treatment who were HIV-infected. |  | NTLP Treatment register linked to, Pre-ART, and ART registers | Indicator C.3.1 [29] |
|  |  | **Numerator:** All other HIV-infected patients enrolled in HIV care and prescribed cotrimoxazole prophylaxis. **Denominator:** All other HIV-infected patients enrolled in HIV care. **Note:** These data were not available for the analysis. |  | Pre ART, and ART registers | Indicator 8a1 [25] |
| 23† | Proportion of HIV-infected, ART eligible patients on lifelong ART | **Numerator:** Number of HIV-infected pregnant women who attended at least one ANC visit who were registered for ART. **Denominator:** Number of HIV-infected pregnant women who attended at least one ANC visit. |  | ANC register linked to ART registers | Indicator 7c [25] |
|  |  | **Numerator:** Number of infants with a positive DNA PCR HIV test result who were registered for ART. **Denominator:** Number of infants with a positive DNA PCR HIV test result. |  | Early Infant Diagnosis laboratory register, ART register | Indicator 29 [25] |
|  |  | **Numerator:** Number of TB-HIV co-infected patients in TB treatment who were registered for ART. **Denominator:** Number of TB-HIV co-infected patients in TB treatment. |  | NTLP register linked to Pre-ART, and ART registers | Indicator C.5.1 [29] |
|  |  | **Numerator**: Number of HIV patient who were registered for ART. **Denominator:** Number of HIV patients who were eligible (according to Uganda National ART Guidelines) to start ART. **Note:** These data were not available for analysis. |  | Pre-ART, and ART registers | Indicator 7c [25] |
| 24 | Proportion of people in HIV care with CD4 test within last 6 months | **Numerator**: Number of HIV patients who had CD4 test within last 6 months. **Denominator:** Number of HIV patients in care. **Note:** These data were not available for analysis. |  | Pre-ART, and ART registers | Indicator 9 [25] |
| 25 | Proportion of people in HIV care who were screened for TB | **Numerator:** Number of HIV patients screened for TB at a visit in the last 3 months. **Denominator:** Number of HIV patients in care in the last 3 months**. Note:** These data were not available for analysis. |  | Pre-ART, and ART registers | Indicator 4 [25] Indicator B1.1 [29] |

† Denotes that the indicator was a FLEI that could have been selected as the focus of CQI activities.

Abbreviations: AFB=Acid-fast bacilli, ANC=Antenatal care, ART=Antiretroviral therapy, DNA PCR=Polymerase chain reaction copy of deoxyribonucleic acid, CQI=Continuous Quality Improvement, FLEI= Facility-Level Evaluation Indicator, HIV=Human Immunodeficiency Syndrome, MOH=Ministry or Health, NTLP=National Tuberculosis and Leprosy Program, TB=Tuberculosis
