# Supplementary material for: Improving Facility Performance in Infectious Disease Care in Uganda: A Mixed Design Study with Pre/Post and Cluster Randomized Trial Components
Source: PLoS One. 2014 Aug 18;9(8):e103017. doi: 10.1371/journal.pone.0103017 (PMC4136733; doi:10.1371/journal.pone.0103017)
Supplement: Table S2 — Total sample size and response rate for facility performance indicators by arm and time period. (DOCX) [file pone.0103017.s002.docx]

| **Table S2. Total sample size and response rate for facility performance indicators by arm and time period** | | | | | | | | |
| --- | --- | --- | --- | --- | --- | --- | --- | --- |
|  |  | **% of sample analyzed** | |  | **Arm A** | | **Arm B** | |
|  | **Performance Indicator** |  |  | **Total** | **Time 0** | **Time 1** | **Time 0** | **Time 1** |
|  | Total patients with revised Medical Form 5 | |  | 777,667 | 101,445 | 240,630 | 204,177 | 231,415 |
|  | Number missing age | |  | 24,593 | 6,633 | 4,846 | 8,806 | 4,308 |
|  | Number of observations with age | | 97% | 753,074 | 94,812 | 235,784 | 195,371 | 227,107 |
| 1† | Proportion of outpatients triaged | |  | Facility-months |  |  |  |  |
|  | Expected (36 facilities X 14 months X 2 age groups) | |  | 1,008 | 180 | 324 | 252 | 252 |
|  | Number of observations | | 99% | 1,002 | 176 | 324 | 250 | 252 |
| 2 | Proportion of emergency and priority patients who were admitted, detained, or referred | |  | Facility-months |  |  |  |  |
|  | Number of observations (1,008 expected, see Indicator 1) | | 89% | 895 | 120 | 324 | 207 | 244 |
| 3 | Estimated proportion of emergency patients who received at least one appropriate treatment | |  | Facility-months |  |  |  |  |
|  | Number of observations (1,008 expected, see Indicator 1) | | 82% | 829 | 101 | 307 | 184 | 237 |
| 4† | Proportion of malaria suspects with a malaria test result recorded | |  | Facility-months |  |  |  |  |
|  | Number of observations (1,008 expected, see Indicator 1) | | 99% | 1,000 | 174 | 324 | 250 | 252 |
| 5 | Estimated proportion of malaria cases who received appropriate antimalarial treatment | |  | Facility-months |  |  |  |  |
|  | Number of observations (1,008 expected, see Indicator 1) | | 99% | 1,000 | 174 | 324 | 250 | 252 |
| 6† | Proportion of patients with a negative malaria test result who were prescribed an antimalarial | |  | Facility-months |  |  |  |  |
|  | # Observations (1,008 expected, see Indicator 1) | | 97% | 977 | 164 | 323 | 248 | 242 |
| 7 | Proportion of patients with a positive malaria test result who were prescribed an antibiotic | |  | Facility-months |  |  |  |  |
|  | Number of observations (1,008 expected, see Indicator 1) | | 96% | 968 | 160 | 321 | 247 | 240 |
| 8 | Proportion of pneumonia suspects aged under 5 years assessed for pneumonia | |  | Facility-months |  |  |  |  |
|  | Expected (36 facilities X 14 months X 1 age group) | |  | 504 | 90 | 162 | 126 | 126 |
|  | Number of observations | | 99% | 498 | 85 | 162 | 125 | 126 |
| 9 | Estimated proportion of patients aged under 5 years diagnosed with pneumonia who received appropriate antibiotic treatment | |  | Facility-months |  |  |  |  |
|  | Number of observations (504 expected, see Indicator 8) | | 98% | 494 | 82 | 161 | 125 | 126 |
| 10† | Proportion of TB suspects with a first AFB smear result | |  | Facility-months |  |  |  |  |
|  | Number of observations (1008 expected, see Indicator 1) | | 96% | 965 | 163 | 313 | 243 | 246 |
| 11† | Estimated proportion of patients with AFB smear negative results who received empiric treatment for acute respiratory infection | |  | Facility-months |  |  |  |  |
|  | Number of observations (504 expected, see Indicator 8) | | 85% | 429 | 69 | 147 | 110 | 103 |
| 12 | Proportion of AFB positive patients prescribed initial TB treatment or referred for TB care | |  | Patients |  |  |  |  |
|  | All AFB positive patients in NTLP laboratory register within date range | |  | 878 | 205 | 301 | 167 | 205 |
|  | Denominator used in analysis | | 87% | 764 | 171 | 267 | 141 | 185 |
| 13† | Proportion of new TB patients with a follow-up AFB smear at 2 months | |  | Patients |  |  |  |  |
|  | All new TB patients in NTLP treatment register within date range (Jan 2009 - Dec 2010) who did not transfer-in after second month of treatment | |  | 2,601 | 787 | 456 | 939 | 419 |
|  | Denominator used in analysis | | 86% | 2,224 | 673 | 418 | 779 | 354 |
| 14 | Proportion of new TB patients with treatment success | |  | Patients |  |  |  |  |
|  | All new TB patients in NTLP treatment register within date range (Jan 2009 - Dec 2010) who did not transfer out | |  | 2,333 | 709 | 399 | 853 | 372 |
|  | Denominator used in analysis | | 74% | 1,645 | 487 | 320 | 563 | 275 |
| 15 | Proportion of TB Patients with an HIV test result recorded | |  | Patients |  |  |  |  |
|  | All TB patients in NTLP treatment register within date range | |  | 1,671 | 283 | 504 | 435 | 449 |
|  | Denominator used in analysis | | 100% | 1,668 | 280 | 504 | 435 | 449 |
| 16† | Proportion of patients with an HIV test result recorded | |  | Facility-months |  |  |  |  |
|  | Revised Medical Form 5 expected (36 facilities X 14 months X 3 age groups) | |  | 1,512 | 270 | 486 | 378 | 378 |
|  | Number of observations - Outpatient subgroup | | 99% | 1,502 | 263 | 486 | 375 | 378 |
|  | Number of observations - TB Suspect subgroup | | 86% | 1,305 | 218 | 421 | 328 | 338 |
|  | ANC register expected (35 Facilities * 14 months) | |  | 490 | 85 | 153 | 126 | 126 |
|  | Number of observations - Pregnant women | | 99% | 486 | 83 | 152 | 125 | 126 |
|  | Number of observations - Partners of pregnant women | | 99% | 486 | 83 | 152 | 125 | 126 |
| 17 | Proportion of HIV-exposed infants with an HIV test result recorded | |  | Patients |  |  |  |  |
|  | Number of births expected among unique ANC women (de-duplicated across visits). Includes multiple children of same ANC woman in Early Infant Diagnosis register. Time 0 = No visit in Time 1 Time 1= Had at least one visit in Time 1 | |  | 3,112 | 428 | 939 | 810 | 935 |
|  | Denominator used in analysis | | 98% | 3,048 | 421 | 900 | 809 | 918 |
| 18 | Proportion of HIV-infected pregnant women who received any ART | |  | Patients |  |  |  |  |
|  | Number unique ANC women (de-duplicated across visits) whose fetus has gestational age greater than 28 weeks. Includes multiple children of same ANC woman in Early Infant Diagnosis register. | |  | 1,943 | 232 | 558 | 522 | 631 |
|  | Denominator used in analysis | | 79% | 1,544 | 176 | 462 | 378 | 528 |
| 19 | Proportion of HIV-infected pregnant women and infants who received ART at delivery | |  | Patients |  |  |  |  |
|  | HIV-infected women in Maternity register | |  | 1,495 | 242 | 395 | 392 | 466 |
|  | Denominator used in analysis | | 89% | 1,334 | 211 | 351 | 359 | 413 |
|  | Live births among HIV-infected women in Maternity register | |  | 1,485 | 239 | 393 | 391 | 462 |
|  | Denominator used in analysis | | 90% | 1,331 | 210 | 350 | 359 | 412 |
| 20 | Proportion of HIV-infected pregnant women that started contraception after delivery | |  | Patients |  |  |  |  |
|  | Number of unique HIV positive women in Post Natal Care register (de-duplicated across visits) Time 0 = No visit in Time 1 Time 1 = At least one visit in Time 1 | |  | 351 | 48 | 123 | 92 | 88 |
|  | Denominator used in analysis | | 60% | 212 | 28 | 44 | 67 | 73 |
| 21† | Proportion of HIV-infected patients enrolled in HIV care | |  | Patients |  |  |  |  |
|  | Number of unique HIV-infected women in ANC register (de-duplicated across visits) | |  | 3,105 | 427 | 938 | 808 | 932 |
|  | Denominator used in analysis | | 100% | 3,103 | 427 | 937 | 807 | 932 |
|  | Number of unique infants in Early Infant Diagnosis register | |  | 1,029 | 125 | 348 | 286 | 270 |
|  | Estimated number who were HIV-infected | |  | 85 | 10 | 40 | 13 | 22 |
|  | Denominator used in analysis | | 91% | 77 | 9 | 37 | 11 | 20 |
|  | All TB Patients in NTLP treatment register within date range, same as Indicator 15 | |  | 1,671 | 283 | 504 | 435 | 449 |
|  | Estimated number who were HIV-infected | |  | 767 | 127 | 218 | 218 | 204 |
|  | Denominator used in analysis | | 90% | 687 | 113 | 206 | 186 | 182 |
| 22† | Proportion of HIV-infected patient and HIV-exposed infants on cotrimoxazole | |  | Patients |  |  |  |  |
|  | Number of unique HIV-infected women in ANC register (de-duplicated across visits), same as Indicator 21 | |  | 3,105 | 427 | 938 | 808 | 932 |
|  | Denominator used in analysis: Number with cotrimoxazole status recorded at least once | | 78% | 2,419 | 312 | 798 | 562 | 747 |
|  | Number of unique HIV-infected women in ANC register (de-duplicated across visits), same as Indicator 21 | |  | 3,105 | 427 | 938 | 808 | 932 |
|  | Denominator used in analysis of HIV-exposed infants: Number of unique HIV-infected women in ANC register with correct arm and time period | | 98% | 3,047 | 427 | 898 | 807 | 915 |
|  | All TB patients in NLTP treatment register within date range, same as Indicator 15 | |  | 1,671 | 283 | 504 | 435 | 449 |
|  | Estimated number who were HIV-infected | |  | 767 | 127 | 218 | 218 | 204 |
|  | Denominator used in analysis | | 77% | 590 | 99 | 169 | 164 | 158 |
| 23† | Proportion of HIV-infected, ART eligible patients on lifelong ART | |  | Patients |  |  |  |  |
|  | Number of unique HIV-infected women in ANC register (de-duplicated across visits), same as Indicator 21 | |  | 3,105 | 427 | 938 | 808 | 932 |
|  | Denominator used in analysis | | 100% | 3,095 | 420 | 937 | 806 | 932 |
|  | Number of unique infants in Early Infant Diagnosis register, same as Indicator 21 | |  | 1,029 | 125 | 348 | 286 | 270 |
|  | Estimated number who were HIV-infected | |  | 85 | 10 | 40 | 13 | 22 |
|  | Denominator used in analysis | | 91% | 77 | 9 | 37 | 11 | 20 |
|  | All TB Patients in NTLP treatment register within date range, same as Indicator 15 | |  | 1,671 | 283 | 504 | 435 | 449 |
|  | Estimated number who were HIV-infected | |  | 767 | 127 | 218 | 218 | 204 |
|  | Denominator used in analysis | | 90% | 687 | 113 | 206 | 186 | 182 |

† Denotes that the indicator was a FLEI that could have been selected as the focus CQI activities.

Abbreviations: AFB=Acid-fast bacilli, ANC=Antenatal care, ART=Antiretroviral therapy, CQI=Continuous Quality Improvement, FLEI= Facility-Level Evaluation Indicator, HIV=Human Immunodeficiency Syndrome, NTLP=National Tuberculosis and Leprosy Program, TB=Tuberculosis
